# Sensorimotor Control in Dystonia

**DOI:** 10.3390/brainsci9040079

**Published:** 2019-04-11

**Authors:** Phillip Desrochers, Alexander Brunfeldt, Christos Sidiropoulos, Florian Kagerer

**Affiliations:** 1 Dept. of Kinesiology, Michigan State University, East Lansing, MI 48824, USA; desroc13@msu.edu (P.D.); brunfeld@msu.edu (A.B.); 2 Dept. of Neurology and Ophthalmology, Michigan State University, East Lansing, MI 48824, USA; Christos.Sidiropoulos@hc.msu.edu; 3 Neuroscience Program, Michigan State University, East Lansing, MI 48824, USA

**Keywords:** movement disorders, sensorimotor control, inhibition, integration, focal dystonia

## Abstract

This is an overview of the sensorimotor impairments in dystonia, a syndrome characterized by sustained or intermittent aberrant movement patterns leading to abnormal movements and/or postures with or without a tremulous component. Dystonia can affect the entire body or specific body regions and results from a plethora of etiologies, including subtle changes in gray and white matter in several brain regions. Research over the last 25 years addressing topics of sensorimotor control has shown functional sensorimotor impairments related to sensorimotor integration, timing, oculomotor and head control, as well as upper and lower limb control. In the context of efforts to update the classification of dystonia, sensorimotor research is highly relevant for a better understanding of the underlying pathology, and potential mechanisms contributing to global and regional dysfunction within the central nervous system. This overview of relevant research regarding sensorimotor control in humans with idiopathic dystonia attempts to frame the dysfunction with respect to what is known regarding motor control in patients and healthy individuals. We also highlight promising avenues for the future study of neuromotor control that may help to further elucidate dystonia etiology, pathology, and functional characteristics.

## 1. Introduction

Dystonia is a complex movement disorder characterized by irregular and involuntary movement patterns and contraction of agonist and antagonist muscles leading to twisted postures with or without a tremulous component [1]. These contractions can be sustained or intermittent and can affect a wide range of muscles and joints. Due to its complex etiology, dystonia can present focally (e.g., cervical dystonia, focal hand dystonia), multifocally, segmentally, or be generalized throughout the body. Such varying presentations make dystonia difficult to diagnose and treat.

In each of these dystonia types, impairment in neuromotor control is observed. In recent decades, advances in understanding the pathophysiology of dystonia and the ability to test motor impairment with advanced technology have allowed for better identification and quantification of sensorimotor dysfunction. This research is of great importance for understanding the functional aspects of dystonia to (1) describe and categorize the impairment with the goal of easier and more accurate identification and (2) gain greater insight into the underlying sensorimotor dysfunction. In this review, we provide an overview on the current state of the dystonia literature with respect to sensorimotor control in humans across dystonia subtypes, with the goal of further elucidating dystonia etiology, identifying areas for potential sensorimotor control research in dystonia, and providing clinicians further means of identifying motor impairment due to dystonia. 

## 2. Impairments in Sensation and Perception

### 2.1. Abnormal Sensory Discrimination and Integration

A large body of evidence suggests sensory impairment in dystonia, which has been reviewed in greater detail elsewhere [2,3,4]. In the 1990s, efforts were made to chart overlap of body regions in the primary sensory cortex (S1) based on the hypothesis that finger representations lacked the clear somatotopic organization typically found in healthy individuals. This phenomenon was first described in non-human primates [5]. Later, Bara-Jimenez and colleagues [6] were among the first to identify abnormal sensory activation within the primary somatosensory cortex (S1) in focal hand dystonia (FHD) in humans. The authors used a high-density electroencephalography (EEG) system to localize the source of neural signals in response to electrical stimulation of the fingers. They found significant overlap in these somatosensory evoked potentials (SEPs) in focal hand dystonia patients, but not in healthy individuals, suggesting that somatotopic organization of the hand areas in S1 was degraded in individuals with FHD due to plasticity-mediated changes. These findings were corroborated using trans-cranial magnetic stimulation (TMS) and magnetoencephalography (MEG) to localize activations of hand areas in M1 and S1, showing development of overlapping corticomotor and somatotopic representations of the hand [7,8]. The identification of S1 abnormalities led to the investigation of spatiotactile and temporal discrimination tasks, which show impaired performance across a variety of dystonia subtypes [9,10,11,12,13,14,15,16]. 

Importantly, in addition to impairments of sensation or perception, several studies have shown that afferent proprioceptive information may not integrate correctly in the sensorimotor system in dystonia [17]. One approach to assess the integration of sensory input and motor output is the use of tendon vibration. In healthy individuals, vibration changes muscle spindle activation, inducing muscle spindle output consistent with muscle stretch, resulting in compensatory contraction of the vibrated muscle via the tonic vibration reflex [18]. Grünewald and colleagues [19] were the first to demonstrate that patients with FHD were impaired in the perception of the amount of limb movement due to the tonic vibration reflex. Participants could not accurately mirror the movement of the vibrated limb with the contralateral limb, suggesting a deficit in movement perception. The impairment was also present when muscle vibration produced the illusion of movement alone, and was generalized to other muscle groups [20,21]. In cervical dystonia (CD), vibration of neck muscles produced smaller changes in posture and sway than in healthy controls [22], but postural responses could be rescued by the application of a sensory trick, which alleviates dystonic symptoms in some patients [23]. Other evidence suggests that the abnormal tonic vibration reflex is rescued by muscle fatigue in patients with CD [24] and may be a promising endophenotypic marker for idiopathic focal dystonia due to its heritability [25]. Abnormal integration is further supported by findings of deficits in kinesthesia when the fingers were passively moved [26] and of reductions in the effectiveness of inhibitory transcranial magnetic stimulation (TMS) conditioning pulses in the periphery on SEPs [27]. Together, these findings point to compromised sensory systems in dystonia. 

### 2.2. Impairment of Spatial Perception and Reference Frames

Impairment of proprioception and its integration with reference frames appears to be present in dystonia, particularly in individuals with CD, affecting the perception of the body’s orientation in its environment. Findings suggest that patients’ allocentric reference frame (i.e., spatial representation of one object to another) is intact, whereas the egocentric reference frame (i.e., spatial representation of objects to the body) is affected. CD patients show impairments when asked to align objects to certain perceived spatial requirements [28]. Furthermore, CD patients have difficulty indicating the subjective ‘straight ahead’. Anastasopoulos and colleagues [29] found that when the body and head were rotated together, patients were able to accurately judge straight ahead in a typical head-centered reference frame. However, when head or trunk rotations were incongruent, visual straight ahead was shifted towards the trunk. This suggests that in the absence of reliable proprioceptive feedback from the neck, patients shifted their egocentric reference frame from the head to the trunk. These findings have been corroborated and extended by others [30,31]. 

Along these lines, others have found increased dependence on allocentric representations. Müller and colleagues [32] found that CD patients were impaired in moving a laser pointer to the straight-ahead position, but non-body centered spatial perception was unimpaired. Similarly, Ploner and colleagues [33] used an egocentric/allocentric spatial memory task to probe internal representations. While CD patients were unimpaired in memory for both egocentric and allocentric conditions, they found that patients used a purely allocentric strategy to accomplish both tasks. Both CD and FHD patients were also impaired in mentally rotating images of body parts to determine whether a presented image showed their right or left side [34,35], indicating impairment with respect to egocentric space, and thus, egocentric reference frames.

### 2.3. Sensory Tricks: Transient Sensory Changes May Modulate Motor Output

Related to atypical sensory function and sensorimotor integration is a common observation, particularly in focal dystonias, of the presence of ‘sensory tricks’ that can transiently quiet dystonic symptoms [36]. Early work using positron emission tomography (PET) scans in CD patients performing their sensory trick demonstrated increased brain activity on the ipsilateral side of the head rotation and decreased activation on the contralateral side [37], suggesting that changes in afferent processing may work to reset the frame of reference of the neck and head for short periods of time. In more recent studies, better discrimination of rapidly presented visual and tactile stimuli was associated with more successful sensory tricks [38]. Activation of sensory networks may stabilize sensorimotor networks by increasing cortical inhibition between involved regions. This is supported by reductions of intrahemispheric facilitation when patients performed their sensory trick [39] and changes in globus pallidus pars interna (GP_i_) activity [40]. This suggests that dormant inhibitory networks are transiently activated with the onset of new sensory information. The existence of sensory tricks in dystonia provides exciting possibilities for studying the role of sensory input to alleviate dystonic symptoms, and merits further investigation.

## 3. Timing

While difficulty in temporal discrimination exists in dystonia, there is also evidence for a more global disruption in central timing mechanisms in dystonia. For example, an interesting study by Filip and colleagues [41] showed that CD patients were impaired when asked to intercept a moving target on a screen by launching a virtual projectile. The authors suggested that predictive central timing mechanisms are defective in CD. Importantly, patients’ performance was not impaired when the predictive timing components of the task were removed. Based on the central role of the cerebellum for timing [42], and because impairment on this task had been noted in patients with cerebellar disorders [43], the authors then showed that a deficit in cerebellar processing could be a candidate for the timing impairment in these patients [44]. The study also revealed lower connectivity with regions in the basal ganglia, including the putamen, pallidum, and caudate. For FHD, findings on timing irregularities are less clear, with one study reporting dysfunction [45] and another demonstrating no impairment [46].

Overall, these findings raise interesting questions. If timing is abnormal, it could be used as a marker of impairment to identify dystonia and/or its severity. Furthermore, impaired timing could inform us more specifically about aspects of neural dysfunction in dystonia. While cerebellar influences on timing have been shown to primarily affect timing variability, basal ganglia networks are also implicated in timing, particularly for error correction [47,48]. Because basal ganglia-cerebellar-thalamocortical loops are hypothesized to be dysfunctional in dystonia [49], evaluation of timing processes in these patients could be an excellent tool to gain more insight as to which parts of the circuitry are affected. 

## 4. Oculomotor and Head Control

### 4.1. Deficits in Ocular Control

Oculomotor control is impaired in dystonia, including saccade control and saccadic adaptation [50,51]. Patients show nystagmic step-patterns of gaze while transitioning from central fixation points to lateral targets during turning movements, as opposed to single-step saccades in controls. Interestingly bradykinesia was also present during trunk turns, which may have caused the nystagmic gaze shifts [50]. These conclusions may be somewhat at odds with the compelling neural integrator hypothesis of cervical dystonia ([52]; for review, [53]), which suggests that abnormal head and eye movements are the result of rapid corrections to a drift in neural circuitry that holds steady a given orientation of the head or eyes. More work is required to further quantify ocular impairments in dystonia and broaden their study to other dystonia subtypes. Dystonia patients are also impaired in classical eyeblink conditioning paradigms that are heavily dependent on olivo-cerebellar function [54], but deficits can be reversed by rapid TMS (rTMS) applied to the cerebellum [55]. Finally, abnormal increases in activation were found in cingulate, primary motor cortex (M1), visual cortex, thalamus, and cerebellum compared to controls in blepharospasm, possibly indicating increased lack of inhibitory control in these networks [56]. Impairments in oculomotor tasks support cerebellar involvement in dystonia, due to high dependence on the cerebellum for oculomotor control. 

### 4.2. Limited Evidence for Deficits in Head Control beyond Cervical Dystonia

Sensorimotor control of the head is almost exclusively studied in individuals with CD. Studies examining the control of head movements in CD have shown that patients demonstrate greater joint position error and consistently overshoot neutral head position [57] and have greater variability in head movements [58]. Dystonia participants also have impaired active reflex modulation of the neck muscles to prevent sudden movements of the head, suggesting that the interaction between voluntary head movements and vestibular reflexes is abnormal [59]. Repositioning the head takes more time in CD [60], possibly due to interruptions of head saccades that would otherwise leave head velocity unimpaired [61]. Vibration of neck muscles seems to restore some proper proprioceptive input and can alleviate deviation of the head [62]. However, even when applied for extended periods of time, this effect remains transient [63]. More recently, Anastasopoulos and colleagues [64] showed that CD patients involuntarily resisted passive head turns, unable to inhibit or integrate proprioceptive head stabilization commands. Since the amount of research focusing on the control of the head is small and focused on CD, more investigation into the control of the head is warranted in all dystonia subtypes.

As mentioned earlier, emerging evidence may point to the dysfunction of neural integrators in the control of head position in CD [52,53]. Shaikh and colleagues [53] have proposed that deficits in head control may be the result of the inability of the CNS to maintain network signaling processes that hold the orientation of the head in a given state, thus requiring fast corrective actions manifesting as the aberrant head movements observed in CD. These authors showed that the patterns of oscillatory head movements, drift velocities, response to restricted visual input, and the direction of drift or recovery movements with respect to the head’s null position are consistent with a dysfunctional neural integrator. Further, this notion is supported by recent work in CD patients that observed decaying firing rates within the globus pallidus and interstitial nucleus of Cajal that were comparable to abnormal muscle activation and head movements [52]. This suggests key involvement of these regions in feedback processing within the neural integrator network. This hypothesis is also supported by work in animal models [65]. Whether the neural integrator hypothesis can explain prolonged contractions observed in CD, in addition to oscillatory head movements, is worthy of additional exploration. It may also be of value to determine whether head control is dysfunctional in other types of dystonia, to better understand control characteristics in different dystonia subtypes.

## 5. Upper Limb Control 

### 5.1. Patterns of Disturbed Motor Control and Internal Model Formation

Surprisingly few studies have examined basic kinematics of upper body movements during tasks commonly used to assess so-called internal models. This widely accepted theoretical concept postulates that in order to produce accurate movements, the neural system integrates information about the motor command and the expected sensory consequences for a movement in a given context [66]. Based on this information, the motor system can then detect discrepancies between the prediction and the observed outcome and make online error corrections. Assessment of movement kinematics offers a window into these processes. In early work, Inzelberg and colleagues [67] found that patients with generalized dystonia of the upper limbs and trunk had asymmetrical velocity profiles and were less accurate during simple reaching movements. The authors also noted that, in dystonia patients, the closed-loop decelerating phase of reaching was more disturbed (as opposed to the feed-forward accelerating phase of movement) and was exacerbated by restricting visual feedback. In the decelerating phase, the motor system must integrate sensory and proprioceptive information into the internal model for movement, adjusting for any state-dependent error. Thus, the authors suggested that in addition to errors caused by involuntary muscle contractions, central processing systems regulating reaching, particularly those involving integration of sensory information with the motor plan, were impaired. 

This research aligns with other work in CD and FHD patients, demonstrating atypical velocity profiles and error control, as well as increased variability, decreased velocity, prolonged movement duration, decreased grip force, and increased co-contraction [68,69,70,71]. Children with dystonia displayed intact speed-accuracy tradeoff behavior in reaching to press buttons of different sizes, but had larger accuracy deficits than typically developing children, likely due to increased noise within the motor system [13]. One very interesting study examined reaching movements before and after botulinum toxin injections in CD patients, showing that reaching movement trajectories, asymmetrical velocity profiles, path lengths, and reversal times were all improved by the treatment [72]. Apart from these studies, there is a clear knowledge gap regarding movement kinematics in dystonia, providing opportunities for more research in motor initiation and termination, feed-forward and feed-back control loops, feedback processing, and internal model formation in different subtypes of dystonia.

### 5.2. Basal Ganglia and Cerebellar Involvement

Robust findings in dystonia pathophysiology over several decades point to abnormalities in basal ganglia function. GP_i_ underactivity is thought to reduce the basal ganglia’s inhibitory influence on the thalamus and thus disinhibit cortical motor areas, resulting in hyperkinetic movements [73]; for review, see [74]. Stimulation of the GP_i_ via deep brain stimulation (DBS) has been shown to be a promising method of treating severe cases of dystonia, normalizing oscillatory activity between the basal ganglia, cortex, and cerebellum [75,76,77]. Basal ganglia activation has also been shown to remain at increased levels following finger tapping tasks, suggesting lack of post-movement inhibition of motor systems [78]. This is also supported by studies examining movement-related cortical potentials in FHD, which are associated with the release of inhibitory influences on motor cortical activity during movement preparation [79,80]. Surprisingly, very few studies have used tasks that are specifically designed to test motor functions of the basal ganglia in dystonia, such as task switching, action selection, or response inhibition. One study tested whether FHD patients could properly cease an action, and found that patients were worse than controls in inhibiting pre-planned responses [81]. Though the authors stopped short of proposing an underlying neural mechanism for this atypical behavior, this task is likely highly basal ganglia dependent.

More recent research has investigated cerebellar influences in motor control of dystonia, and cerebellar dysfunction has become a key finding in many studies (for review, see [82]). In addition to timing impairments (see above), cerebellar-dependent motor adaptation impairments have also been observed in dystonic individuals, although the results of different studies are equivocal; some studies report disturbances [83,84,85], while others do not [69,86,87]. If abnormal upper limb motor adaptation is intact, this presents an intriguing discrepancy when compared to atypical saccadic adaptation, which also is cerebellum dependent. This suggests that the cerebellum may not be uniformly disrupted in dystonia, or that these adaptation processes rely on distinct and differently affected networks or adaptation strategies. Importantly, both cerebellum and basal ganglia are implicated in both timing and adaptation tasks [48,88]. For example, rapid onset vs. gradual onset of visuomotor perturbations in reaching tasks differentially engage the cerebellum and basal ganglia [89,90,91,92], as do different phases and aspects of motor learning [93,94,95,96]. Interestingly, some studies have suggested that cerebellar abnormalities in dystonia may not be the core source of dysfunction, and that the cerebellum may act in a compensatory nature for dysfunction in other systems [69,97]. Teasing apart the processes by which each brain region contributes to these tasks in dystonia is worth examining. 

### 5.3. Atypical Inter- and Intrahemispheric Communication and Inhibition During Motor Tasks

Communication amongst brain regions, between and within hemispheres, seems to be atypical in individuals with dystonia during motor tasks. For example, the use of TMS has demonstrated lack of local inhibitory control [81,98,99,100,101,102]. Transient suppression of M1 via rTMS allows for improved writing speed and maze completion [103]. Additionally, Hummel and colleagues [104] demonstrated a lack of event-related synchronization in EEG recordings in dystonia participants as compared to healthy controls in a task in which they had to observe but not execute a learned finger tapping sequence. They proposed that underlying neural populations were less adept at the inhibition of the motor command. Abnormal functional connectivity between cerebellum and globus pallidus has also been found in idiopathic dystonia patients [76], further supporting abnormal neural connectivity throughout the sensorimotor network.

A common clinical observation is the presence of motor overflow and mirror movements in dystonia, typically observed in FHD, but also in other dystonia subtypes. In these movements, action of one part of the body causes involuntary activations in another effector [105,106]. These movements are thought to be the result of dysfunctional intra- and intercortical inhibition. Within M1, surround inhibition, which reduces co-activation of neurons controlling neighboring muscles, has been found to be reduced in dystonia patients and may contribute to ipsilateral overflow [107]. Interestingly, a recent study suggests that surround inhibition may be normalized in focal hand dystonia following paired associative stimulation of the periphery [108]. Interhemispheric inhibition was also substantially decreased in patients with mirror movements compared to controls and patients who did not display mirror movements and is correlated with disease severity and the presence of mirror movements [109,110]. Others have shown abnormal motor unit synchronization and motor overflow of a central command [111], using intramuscular EMG in patients with FHD. Taken together, the atypical intra- and interhemispheric inhibition may allow for increased sharing of motor information between the hemispheres, and thus leave dystonia patients more susceptible to interference between limb movements. Interestingly, some evidence suggests that muscle synergies, or modules of commonly co-activated muscles, may be intact in dystonic children [112,113]. This suggests that despite motor impairments, the nervous system retains functionally coupled muscle activity during voluntary movement. 

## 6. Lower Limb Control

### 6.1. Impairment of Gait and Balance in Several Dystonia Subtypes

In comparison with studies of the upper body, investigations of lower limb control in dystonia are less numerous. Early examinations of postural control examined the onset of sway due to vibration of neck muscles and changes in place stepping action [22,114]. More recently, Hoffland and colleagues [97] used split-belt treadmill walking and motion capture analysis to explore gait adaptation, a highly cerebellar dependent task. Patients with CD were not different than controls in gait adaptation, but patients with blepharospasm and FHD adapted slower and had greater asymmetry in step length. This interesting dichotomy within focal dystonias and with respect to upper limb adaptation experiments warrants replication and further investigation and should motivate further study of gait in focal dystonia. Meanwhile, Barr and colleagues [115] tested patients on several different tasks, including walking on a pressure-sensing walkway, timed up and go test, balance, and hand and foot reaction times. They found that in patients, walking speed was slower, step variability was greater, and patients spent more time in double stance phase during locomotion. Furthermore, balance was more variable in CD patients as compared to controls, particularly with visual feedback restricted. Postural sway while seated was also greater in CD patients, and was exacerbated by tremor, indicating robust deficits in postural control in these patients [116]. These studies demonstrate numerous functional deficits in dystonic gait and posture that deserve greater research attention.

### 6.2. Deep Brain Stimulation in Dystonia: A Possible Link to Bradykinesia?

As opposed to direct study of lower limb sensorimotor control in dystonia, an important new line of inquiry has begun examining the onset of parkinsonisms associated with deep brain stimulation of the GP_i_ (DBS-GP_i_). DBS-GP_i_ is a highly successful treatment used in cases in which medication and botulinum toxin injections do not alleviate dystonic symptoms [77]. However, there have been recent reports of bradykinesia and hypokinetic movements being observed in patients receiving DBS treatment. Schrader and colleagues [117] tracked patients having undergone DBS surgery and assessed reports of novel gait disturbances. In 8.5% of cases, novel gait disorders had occurred, characterized by gait freezing, shuffling steps, difficulties turning, and bradykinesia. Similarly, Berman and colleagues [118] retrospectively characterized parkinsonian impairments in DBS-GP_i_ patients, and determined that 82% of patients reported slowing of other body movements, including micrographia and bradykinesia. These findings have been corroborated by others [119], and show that hypokinetic movements can be observed in dystonia as a result of DBS. It is important to note that even with the appearance of parkinsonian symptoms, dystonic symptoms were still successfully mitigated by DBS. Better quantitative analysis of these new symptoms may provide greater insight into network disorders within the basal ganglia in dystonia and provides interesting avenues of study for elucidating factors behind gait disturbance in Parkinson’s disease. 

## 7. Pharmacological Approaches and Their Effects on Sensorimotor Control

A variety of pharmacological approaches have been used in dystonia, all of which are constrained by limited efficacy and side effect profile. Anticholinergics are typically used with modest benefit, and are thought to restore synaptic plasticity in the striatum mostly through M1-receptor antagonism [120]. However, they oftentimes lead to troublesome side effects, such as cognitive blunting, hallucinations, xerostomia, and constipation. Spasmolytics, such as baclofen, are also used and act mainly on GABA receptors in the brain and spinal cord, particularly GABA-b receptors. Enhanced GABA-ergic transmission in the brain is thought to lead to increased inhibition in the basal ganglia-sensorimotor cortex loop. Finally, botulinum toxin injections not only act as a local muscle relaxant by chemically denervating extrafusal muscle fibers, but also have an effect on the sensory input by affecting the intrafusal muscle fibers, representing a form of long-lasting sensory trick and modulating CNS activity [121,122]. Side effects typically include weakness of muscles in proximity to the injection site.

Due to the upstream effects of botulinum toxin, many researchers have sought to categorize how botulinum toxin might modulate neural activity and sensorimotor control. Early work showed that speed and accuracy of handwriting improved dramatically following botulinum toxin injection [123,124]. Later, studies using more robust kinematic measures showed that botulinum toxin modulated the voluntary movement of dystonic and non-dystonic segments, with restored movement patterns approaching those of healthy controls [60,72,125]. Botulinum toxin treatment also modulates involuntary, reflexive movements, albeit not always towards that of controls [126,127], suggesting that the effects of botulinum toxin act at both higher and lower motor centers within the CNS. Cortical mapping studies have demonstrated that botulinum toxin produces more normal patterns of cortical topographical activation [8,128,129], though differences between patients and healthy controls may still be evident in the basal ganglia [130]. Thus, patients can show improved sensory discrimination following botulinum toxin injection [131]. Taken together, it is clear that botulinum toxin treatment not only successfully relieves dystonic symptoms but also produces substantial changes within the CNS. Continued investigation of botulinum toxin’s effects on sensorimotor control is advantageous for understanding its underlying mechanisms. In particular, using movement tasks that probe specific components of motor planning, movement execution, or feedback integration may yield additional insights into its function and atypical neural processes in dystonia.

## 8. Discussion

We have examined a range of research in dystonia from a sensorimotor control viewpoint, spanning sensory and perceptual systems, timing, head and ocular control, upper and lower limb control, and pharmacological effects. However, there exists a key dichotomy in sensorimotor control research in dystonia. The majority of studies can be parsed into those that examine neural integration of sensorimotor information (see Table 1) or those that examine lack of inhibition (see Table 2). However, few have examined dystonia as both a dysfunction of inhibition and integration. This raises interesting questions as to the interconnectedness of these two areas. Using a two-pronged approach—investigating sensorimotor integration/adaptation and the neurophysiology underlying intracortical and interhemispheric inhibition may afford more testable predictions than the current, very broad conceptual description of dystonia as a network dysfunction. In other words, this could provide a framework by which networks can be evaluated for specific dysfunction of neural integration and inhibition. As such, we promote the notion here that dystonia is best characterized as a dysfunction of both integration and inhibition within basal ganglia-cerebellar-thalamocortical circuitry.

The concept of internal models in sensorimotor control is of direct relevance to dystonia research. It appears that in dystonia, the comparison between the predicted sensory feedback and observed sensory input is disrupted. Whether this is due to errors in the generation of the efference copy or distorted sensory information is an open question. It is clear that afferent signals from the periphery are not correctly integrated in the CNS. The reflex pathway remains functional, as shown by contraction of vibrated muscles. However, the lack of reflex perception and influence on subsequent movement may reflect the inability of the CNS to successfully incorporate incoming afferent feedback into internal models, a function thought to be highly dependent on communication between the posterior parietal cortex, premotor areas, basal ganglia, and cerebellum [94,132,133,134]. Emerging evidence in the dystonia literature throughout the past decade points increasingly to dysfunctional cerebellar-basal ganglia-thalamocortical loops as a contributing factor in dystonia [4,49,52,135]. 

It should be noted that while research on sensorimotor control in dystonia has made remarkable progress in recent years, the vast majority of the research has focused on focal dystonia, with much less attention paid to idiopathic generalized, multisegmental, or hemi-dystonia, or secondary dystonia. The cause of this is likely two-fold. First, though epidemiological studies of dystonia vary widely in their reported rates of incidence, it is consistently found that focal dystonias are significantly more common than other subtypes [109,110,111], resulting in easier recruitment and study. Second, the process of quantifying movement in multisegmental and generalized dystonia is hampered by the presence of the disorder itself, whereas in focal dystonia, many movements are relatively intact and can be quantified. Creative solutions to this problem would be extremely beneficial for understanding sensorimotor control in non-focal dystonia. Additionally, such studies would be very informative in parsing phenomenological differences between focal and non-focal dystonia subtypes. Nevertheless, extant research reviewed in this paper suggests that sensorimotor integration problems and atypical inhibition are features present across dystonia subtypes, and could be addressed meaningfully through systematic meta-analyses or targeted future work.

Quantifying motor control functions could provide valuable insights into etiology and pathology, and may also provide possible vectors for treatment or rehabilitation strategies. Tests of action selection, task switching, motor planning, trajectory control, and forms of adaptation are notably absent from motor control literature in individuals with dystonia. It has been well documented that performance in such tasks is impaired in other basal ganglia disorders like Parkinson’s disease [136,137,138]. How dystonia patients perform in these domains is of important clinical value, allowing for faster detection of dystonic symptoms, more discrete classification of impairments, and better delineation of underlying sensorimotor deficit in a specific patient. For researchers, better quantification of impairment in dystonia can inform greater understanding of sensorimotor networks involved in the syndrome. Furthermore, the ever-growing body of literature implicating numerous neural regions in dystonia presents opportunities for further identifying networks involved in motor control. In this context, dystonia presents an opportunity for designing experiments that address still open questions in motor control such as internal model formation, regional contribution to motor learning, and control of degrees of freedom in the motor system.

## 9. Conclusions

Evaluations of sensorimotor control in dystonia have allowed for great leaps forward in understanding the phenomenology and etiology of the syndrome. However, further steps can be taken to quantify impairments, categorize motor control functions that are intact, and localize dysfunction to specific neural structures and networks. Furthermore, sensorimotor control may be altered for different dystonia subtypes. Understanding where these differences lie is a valuable undertaking and could lead more well-defined treatment regiments for different subtypes or elucidate specific neural sources of pathology. Modern technologies in robotics, motion capture, accelerometry, imaging, and signal processing should be brought to bear on these important questions.

## Figures and Tables

**Table 1:** References addressing sensorimotor integration in patients with cervical dystonia (CD), focal hand dystonia (FHD), benign essential blepharospasm (BEB), and DYT1 dystonia (DYT1). The number after the year reflects position in the reference list. Under Participants, N_D_, N_C_, and N_O_ respectively denote dystonia, control, and other non-dystonic movement disorder sample sizes, R denotes age range, M denotes mean (standard deviation), and D denotes duration of symptoms, all in years.

Author/Year	Participants	Type	Technique / Design	Key Findings
Anastasopoulos, 1998 [29]	N_D_: 10, N_C_: 12, M: 43.0 (13.9), D: 4–16	CD	Perception of ‘visual straight ahead’ (VSA)	VSA shifted to trunk under head/trunk misalignment. Mechanisms suggested: (i) central compensation restoring VSA, (ii) reference frame shift to more stable trunk coord. system
Anastasopoulos, 2003 [58]	N_D_: 12, N_C_: 12, M: 43.8 (10.7), D: 6.1 (3.4)	CD	Neutral head position estimation after head/trunk displacement	Patients use neck proprioception, but lack head posture knowledge, suggesting an offset of a non-sensory setpoint.
Anastasopoulos, 2013 [50]	N_D_: 8, N_C_: 10, R: 42–72, D: 2–10	CD	Peripheral target foveation and trunk kinematic assessment	Prevalence of hypometric gaze saccades and trunk bradykinesia in neck dystonia.
Anastasopoulos, 2014 [64]	N_D_: 13, N_C_: 23, R: 20–61, D: 3–16	CD	Measured resistive torques to passive head/trunk/head+trunk movements	Resistive torques higher in patients than controls and independent of torticollis direction, suggesting impaired proprioceptive feedback.
Avanzino, 2013 [45]	N_D_: 14, N_C_: 17, M: 42.3 (12.3), D: 9.6 (7.4)	FHD	Temporal expectation task: video of hand motion or inanimate obj.	More timing error in patients when viewing hand motion vs. inanimate object motion, suggesting planning deficits.
Avanzino, 2018 [85]	N_D_: 20, N_C_: 17, M: 60.3 (11.5)	CD	Catching a ball with unpredictable mass	Adaptation to heavier mass similar between patients and controls, but the anticipatory adjustment to impact reduced for patients, suggesting cerebellum’s role in predictive control is abnormal in CD.
Bove, 2004 [114]	N_D_: 12, N_C_: 12, M: 59 (15.1), D: 9.4 (5.5)	CD	Postural balance and stepping in place w/wo vibration to neck	Reference frame for body orientation progresses to different egocentric reference as disease advances.
Brugger, 2018 [23]	N_D_: 35, N_C_: 16, Older adults, D: ~16 (12.5)	CD	Quiet stance posture analysis during neck vibration with/without effective sensory trick	Patients with effective sensory trick responded similarly to controls during vibration; those without had little change in posture. Effectiveness of sensory trick may require an intact ability to preserve proprioceptive gain.
De Pauw, 2017 [57]	N_D_: 24, N_C_: 70, No ages listed, D: 13 (8.7)	CD	3D motion tracking of return-to-neutral head position	Larger positional errors in patients than in controls, and tendency to overshoot return to neutral head position.
De Pauw, 2018 [116]	N_D_: 23, N_C_: 36, M: 59.4 (14.6), D: 13 (8.7)	CD	Seated postural control	Postural instability was increased in patients, with center of pressure correlating to impairments in cervical sensorimotor control.
Filip, 2013 [41]	N_D_: 30, N_C_: 30, M: 52 (13.7), D: 3–38	CD	Virtual projectile intercept task	Visual input - predictive motor control integration problem, suggesting impairment to the cerebellar anticipatory timing function and ability to integrate visual and motor information.
Frima, 2003 [24]	N_D_: 21, N_C_: 18, R: 29-72	CD	Tendon vibration inducing illusion of elbow joint movement	Perception of movement increased in patients, suggesting subnormal muscle spindles elasticity.
Frima, 2008 [25]	N_D_: 30, N_C_: 19, R: 29–75, incl. parents, siblings, children	CD	Same as Frima 2003	Higher prevalence of abnormal perception in 1^st^ degree relatives, suggesting trait heritability.
Hoffland, 2014 [97]	N_D_: 26, N_C_: 10, M: 56.5 (8.2)	CD, FHD, BEB	Split-belt gait adaptation with 3D motion capture	Gait adaptation impairment in BEB and FHD, but not CD patients, suggesting different cerebellar pathologies.
Hubsch, 2011 [51]	N_D_: 14, N_C_: 14, M: 31.8 (15.1)	DYT1	Reactive saccade adaptation	Less adaptation in patients than controls, suggesting cerebellar dysfunction in DYT1 dystonia (myoclonus).
Inzelberg, 1995 [67]	N_D_: 8, N_C_: 6, R: 19–43, D: 2–27	non-specific	Temporal and spatial analysis of unimanual reaching w/wo vision	Velocity profiles in patients less symmetric than in controls, longer deceleration phase, similar to PD. Impairments were more prevalent during ‘closed-loop’ control suggesting abnormalities in integrating feedback into subsequent motor commands.
Kägi, 2013 [38]	N_D_: 32, N_C_: not specified, M: 56.4 (9.9), D: 12.5 (8.5)	CD	Temporal discrimination to visual, tactile, and visuotactile stimuli	Visuotactile discrimination improvement with sensory tricks, particularly in patients with shorter disease duration, suggesting a progressive loss of adaptive mechanisms.
Karnath, 2000 [63]	Case study of 48 y/o female	CD	3D tracking of head position before/after vibration	More improvement after vibration than TENS or haptics, suggesting impaired central processing of neck muscle afferents.
Katschnig-Winter, 2014 [69]	N_D_: 12, N_C_: 11, M: 58.8 (9.6), D: 6–36	CD	Center-out reaching: motor reference task, sequence learning, motor adaptation	Higher peak velocities, longer movement times in patients, normal sequence learning and motor adaptation.
Lekhel, 1997 [22]	N_D_: 19, N_C_: 12, M: 33.8, R: 24–49	CD, FHD, BEB	Postural sway analysis, with vibration of neck muscles	Decreased postural sway in patients, possibly due to vestibular signal - neck muscle spindle signal integration.
Müller, 2004 [32]	N_D_: 28, N_C_: 28, M: 49.5 (14.3), D: 0.5–43	CD	Subjective straight-ahead task, BORB battery, VOSP battery	Intact allocentric but compromised egocentric spatial abilities in patients; reliance on proprioceptive neck inputs.
Naumann, 2000 [37]	N_D_: 10, R: 28–76, D: 1–19	CD	PET recording in response to sensory trick application	Sensory tricks normalizing head position shift egocentric midline reference to opposite side of head turn, decreasing M1 activation.
Pelosin, 2009 [72]	N_D_: 10, N_C_: 10, M: 50.5, R: 35-65, D: 1-10	CD	Center-out reaching before/after botulinum toxin treatment	Botox improved spatiotemporal control of reaching, possibly improving proprioceptive feedback by relaxation of muscle spindles.
Putzki, 2006 [26]	N_D_: 23, N_C_: 13, R: 42–64, D: 0.5–24	CD, BEB	Passive finger movement detection and discrimination	Patients were less sensitive to movement, with poorer directional discrimination, suggesting contribution of defective sensory processing to dystonic symptoms.
Rome, 1999 [20]	N_D_: 24, N_C_: 18, N_O_: 21, R: 30–77, D: 1–33	CD, FHD	Arm position matching, with/without tendon vibration	Contralateral joint position perception impaired in dystonia, but not PD. Botox injections did not recover function.
Sadnicka, 2018 [84]	N_D_: 10, N_C_: 12, M: 43.9 (14.3), D: 2–58	DYT1	Center-out reaching with visuomotor perturbation	Increased baseline task-dependent variability predicted poor adaptation in patients. Specifically, variability in feedforward component of movement was most predictive, suggesting unwanted noise affects planning, but not online corrective actions.
Sedov, 2019 [52]	N_D_: 12, R: 22–68, D: 2–17	CD	In-vivo single-unit neuron recording in basal ganglia and EMG of trapezius	Malfunction of neural integrator results from impairments to cerebellar, basal ganglia, and feedback converging on integrator. Asymmetry in pallidal activity correlated with degree and direction of head turning.
Vacherot, 2007 [31]	N_D_: 12, N_C_: 11, M: 63 (4.7)	CD	Balance testing, assessing subjective visual vertical	Whole body stabilization not affected in patients, but head stabilization reliant on referencing the trunk.
van der Steen, 2014 [46]	N_D_: 15, N_C_: 15, M: 36.5 (12), D: 1–20, prof. musicians	FHD	Temporal perception and motor task battery	Musician’s dystonia not associated with sensory deficits; likely a highly task-specific disorder.
Yoneda, 2000 [21]	N_D_: 29, N_C_: 15, M: 57.7, R: 29–79, D: 11.5	CD, FHD, BEB	Arm position matching, with/without tendon vibration	Abnormal perception of tonic vibration reflex in patients suggests abnormal muscle spindle afferent processing. Despite localized motor deficits, authors suggest FHD is a systemic disorder.

**Table 2:** References addressing cortical inhibition/excitation in patients with focal hand dystonia (FHD), cervical dystonia (CD), benign essential blepharospasm (BEB), generalized (gen), or DYT1 dystonia (DYT1). The number after the year reflects position in the reference list. Under Participants, N_D_ and N_C_ respectively denote dystonia and control sample sizes, R denotes age range, M denotes mean (standard deviation), and D denotes duration of symptoms, all in years.

Author/Year	Participants	Type	Technique / Design	Key Findings
Abbruzzese, 2001 [27]	N_D_: 21, N_C_: 16, R: 28–78, D: 1–27	FHD, CD	TMS targeting APB, median nerve stimulation	Inhibitory effect of median nerve stimulation in CD and controls, but not in FHD.
Amadio, 2014 [39]	N_D_: 8, N_C_: 8, R: 30–61	CD	TMS w/wo sensory trick application	Sensory tricks reduced abnormal intracortical facilitation, suggesting improved M1 cortical inhibition.
Antelmi, 2016 [16]	N_D_: 19, N_C_: 19, M: 62.6 (9.2), D: 9.42 (4.7) 3 months post botox injection	CD	SEP recording	S1 disinhibition in CD, compared to healthy controls.
Baker, 2003 [56]	N_D_: 5, N_C_: 5, R: 50–62	BEB	BOLD activation mapping during spontaneous / voluntary blinking	Anterior visual cortex, central thalamus, and superior cerebellum activation larger patients than controls.
Beck, 2008 [107]	N_D_: 16, N_C_: 20, R: 43–72, D: 3–39	FHD	EMG recording of APB during isometric FDI flexion. TMS used to measure inhibition	Patients failed to modulate APB activity during FDI contraction and showed decreased inhibition to APB. This was prominent during movement initiation.
Beck, 2009 [109]	N_D_: 13, N_C_: 12, R: 44–73, D: 3–39	FHD	TMS targeting *abductor pollicis brevis* (APB)	Reduced IHI in mirror dystonia, but not in FHD patients without mirroring.
Blood, 2004 [78]	N_D_: 8, N_C_: 5, R: 31–58	FHD	fMRI during bilateral finger tapping	Higher caudate nucleus, putamen, globus pallidus, and M1 activation in FHD patients than controls.
Gilio, 2003 [98]	N_D_: 10, N_C_: 8, M: 40 (1.3), D: 2–15	FHD, gen	TMS over M1 targeting wrist extensors	No cortical excitability changes, small intracortical inhibition changes in patients; increased excitability / reduced inhibition in controls.
Hoffland, 2013 [55]	N_D_: 19, N_C_: 8, Older adults, D: 13 (7)	CD	Eyeblink conditioning, cerebellar TMS	cTMS improved eyeblink conditioning in CD.
Huang, 2010 [103]	N_D_: 11, N_C_: 9, R: 27–57, age at onset: 9–47	FHD, DYT1	rTMS over dorsal premotor cortex	Suppression of cortical excitability in controls and DYT1 dystonia, but not in FHD. rTMS improved intracortical inhibition and writing function in FHD.
Hubsch, 2013 [83]	N_D_: 21, N_C_: 25, M: 42.9 (14.3), D: 0.5–31	FHD	TMS over M1 and cerebellum. Correlation with adaptation task.	No sensorimotor plasticity modulation; reduced motor adaptation in patients, more robust cerebellar inhibition.
Hummel, 2002 [104]	N_D_: 6, N_C_: 18, R: 29–68	FHD	EEG and TMS during activation or inhibition of motor program	Inhibition of learned motor program was associated with increase in alpha oscillations in controls but not in patients. This suggests increased oscillation is a mechanism by which motor programs are inhibited.
Ridding, 1995 [99]	N_D_: 15, N_C_: 8, M: 47 (13)	FHD	TMS over M1 targeting *first dorsal interosseous* (FDI)	Decreased inhibition of hemisphere controlling the dystonic hand in patients. Similar excitability in patients and controls.
Sitburana, 2009 [106]	N_D_: 30, N_C_: 40, M: 51 (11.8), D: 9.7 (7.4)	FHD	Handwriting analysis; repetitive hand tasks	More motor overflow in patients than controls. Mirror overflow most prevalent, followed by ipsi- and contralateral.
Terranova, 2018 [108]	N_D_: 8, N_C_: 8, R: 31–66	FHD	Paired associative stimulation (PAS), SEP recording	While facilitation was larger for patients and spatial specificity was lost, inhibition was similar between patients and controls.
Tinazzi, 2000 [15]	N_D_: 10, N_C_: 10, M: 45.3 (8.1)	FHD, gen	SEP recording	SEP disinhibition, suggesting impaired afferent input gating, affecting motor excitability.
